# Reframing Maslow to Comprehensively Consider the Needs of All Trainees

**DOI:** 10.5334/pme.1886

**Published:** 2026-01-28

**Authors:** Kara Alcegueire, Rachel B. Levine, Arianne Teherani, Scott M. Wright

**Affiliations:** 1 Hospital Medicine, Johns Hopkins Bayview Medical Center, Johns Hopkins University School of Medicine, Baltimore, Maryland, USA; 2 Division of General Internal Medicine, Johns Hopkins Bayview Medical Center, Johns Hopkins University School of Medicine, Baltimore, Maryland, USA; 3 Center for Faculty Educators, University of California, San Francisco School of Medicine, San Francisco, California, USA

## Abstract

The medical education system is not designed to recognize and support the nuanced needs of medical trainees from historically marginalized groups. Consequently, many from this cohort must persevere through their medical training with various unmet needs. Within the medical education system, there have long been systemic barriers, or structural inequities, that make training more difficult for trainees from historically marginalized groups; the situation is unfair and can translate into unequal educational outcomes. Regrettably, the convention of ignoring the impact of structural barriers and resultant inequities for trainees from historically marginalized groups may lead to assumptions of innate deficiencies. This deficiency discourse obscures the impact of structural barriers on these individuals’ learning, well-being, and academic outcomes. In this paper, we present a novel framework to bring attention to these challenges. This framework emerges from Maslow’s ‘Hierarchy of Needs’ theory to emphasize the importance of comprehensively considering trainee needs that ultimately support their learning and flourishing. Practically, this modified framework may be used directly by trainees themselves to reflect on their needs, and by educators charged with supporting them to more fully bear in mind the lived experiences of trainees. At the programmatic level, the framework may reveal inequities that may be differentially influential within a cohort – thereby informing efforts to support all trainees.

## Introduction

Medical education systems largely view trainees as a uniform group; this is rooted in the assumption that all trainees will be able to flourish as they progress through the standard curriculum [1,2,3]. This subverts educational equity and undermines certain trainees, especially those from historically marginalized groups whose lived experiences are not practically acknowledged [4,5]. Many trainees, particularly those from historically marginalized groups, experience a lack of appropriate structural support for their identity-specific challenges within the medical education systems. These “structural barriers” can make the learning environment feel intimidating and insensitive [4,5,6]. Compared to their non-historically marginalized peers, many medical trainees with these identities experience structural inequities, systemic differences in outcomes that result from structural barriers and discriminatory systems, that place disadvantages on them [6,7,8,9,10].

The route to becoming a physician is long and demanding for all trainees – regardless of background and identity. However, this course is more daunting for many medical trainees from historically marginalized groups as they try to navigate the path that has been designed for students with significant resources – especially, financial, and social [1,6,11,12,13]. Both intrinsic disadvantages and superimposed burdens make it less likely that some trainees from historically marginalized groups will have their needs adequately met by the standard level of support that is offered to all trainees [1,4,7,12,14]. By comparison, non-historically marginalized trainees encounter fewer stressors, problems, and distractions that pull them away from attending to their professional development; they are simultaneously buffered by having more familial, financial, and social support [1,6]. To address such inequities and improve the support offered to trainees, ideally the medical education system can become safer, less overwhelming, and more inclusive. This will be critical to facilitate the development of all medical trainees so they may flourish [15].

There is extensive evidence of the identity-specific inequities faced by many medical trainees who identify as being from historically marginalized groups – including, but not limited to first-generation (FG), low-income (LI), first-generation low-income (FGLI), historical marginalized racial groups, as well as those with disabilities [1,16,17,18]. For example, low-income trainees may have their learning undermined by financial insecurity while historically marginalized racial trainees can often have their learning impeded by being made to feel like they don’t belong (othering) or due to exposure to discrimination (from peers or educators) and from discriminatory elements embedded within the formal curriculum (learning about ‘ethnic’ diseases in ways that essentialize difference) [1,17,19,20,21,22]. While seemingly minor to those unaffected, such exposures may place significant stress on their development and learning [23].

Academic attainment gaps during medical training for these historically marginalized groups has been described in the literature compared to non-historically marginalized peers [6,9,17]. However, the relationship between structural inequities and lower academic and clinical performance among trainees from these groups has not been well characterized or explored in depth [6,11,17,24]. While it may be difficult to quantify the impact of the structural influences involved, it does not help that there is no shared mental model to describe the structural inequities faced by historically marginalized groups training in healthcare [6,11,17,18,24]. Without this context, systems and educators may fall back on traditional substantiation of meritocracy leading to academic success while also presuming individual deficiency for underperformance [4,25,26,27]. These narratives falsely presume that all trainees have access to comparable resources and systems of support, and they absolve the medical education system of accountability [5,25,26,27]. The deficit discourse stigmatizes trainees from historically marginalized groups and implicitly suggests their inferiority compared to peers [13,18,25,26]. These explanations can instigate mental and emotional harm to struggling medical trainees by treating them as “problems” that require fixing [25,26].

Of practical utility, it would be helpful for the medical education system to have a model for understanding trainees’ lived experiences and the structural inequities that may undermine their learning and academic outcomes. With this, the healthcare workforce might begin to better reflect the general population; without such considerations, the status quo of having relatively few physicians from these groups will continue owing to dynamics including admission practices, rigid institutional structures, and disproportional attrition rates [4]. To this end, this paper introduces a framework based on Maslow’s Hierarchy of Needs (MHN) theory. This novel framework for medical education aims to raise awareness about the links between structural inequities, trainees’ unmet needs, and academic performance. Specifically, this framework brings attention to the structural inequities, which often manifest as unmet needs, that disproportionally impact the learning of many trainees who are from historically marginalized groups [4,5,6]. The framework is the first to use MHN theory to bring attention to the impact of structural inequities on trainees. If successful, the framework may direct educators and programs to better support trainees by taking a more thorough and thoughtful approach to understanding trainees’ needs [4].

## Theoretical Background: Maslow’s Hierarchy of Needs

**Figure 1:**
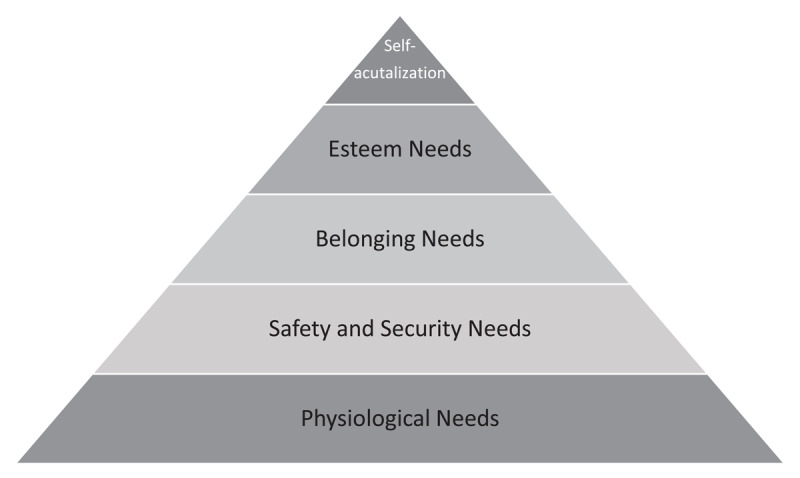
**Schematic representation of Maslow’s Hierarchy of Needs Theory**. Abraham Maslow published his conception of hierarchy of needs – called “A Theory of Human Motivation” in 1943. According to the model, the needs had to be satisfied in a sequential order, starting with physiological needs. When all needs are fulfilled, an individual arrives at self-actualization – believed to be the state in which they can perform at their full potential.

Abraham Maslow conceived his MHN theory to explain human motivations [28]. He posited that multiple needs must be met to excel in complex endeavors – such as becoming a physician. The model proposed that individuals satisfy sequentially ordered needs on their way to success (Figure 1) [29]. In ascending order, his model asserted that need fulfillment progresses through the following –physiological, safety and security, belonging, esteem, and ultimately culminating in self-actualization [28,29].

An individual’s physiological needs are the biological essentials needed to survive, which include access to air, water, food, shelter, and rest. Security and safety needs represent those that afford protect individuals from emotional, psychological, and physical harm. Belonging refers to an individual’s need for connectedness and acceptance from others. Esteem relates to the individual’s need for self-acceptance and recognition from others. Self-actualization ultimately may occur when all needs are met, thereby allowing the individual to realize their fullest potential [28,29].

## Previous Application of Maslow’s Hierarchy of Needs in Medical Education

Over the last 80 years, Maslow’s theory has been applied across multiple fields, including medicine. With respect to learning medicine, previous applications have repurposed the model as a holistic model for supporting physicians – both in graduate medical education (GME) and for those who have completed their training [28,30,31,32,33]. In these papers, it was suggested that attending to belonging needs might reinforce well-being among trainees and faculty physicians [28,30,31,32]. Another paper proposed that MHN might be helpful to consider when supporting the unique needs of international medical graduates (IMG) as they adapt to working in the U.S [33]. The framework described in this paper adapts MHN theory to draw attention to the structural inequities faced by historically marginalized groups during their medical training. By design, the adapted framework allows for the consideration of multiple identities; this represents a novel application of MHN by examining equity through an intersectionality lens.

## Adapting Maslow’s Hierarchy of Needs Theory for Equity Within Medical Education

No two trainees are the same. Medical trainees with distinct identities and backgrounds are likely to have different needs and preferences in how their needs are fulfilled. Medical trainees from historically marginalized groups may face structural barriers and inequities that place them at risk for not having their needs met during their medical training. For practical application within the medical education system that is underfunded and overstretched, we adapted MHN theory to create a novel framework that could be used to encourage trainees, educators, and institutions to consider the holistic support of trainees through the lens of equity.

In considering the relationship between trainees’ needs and their academic performance within the medical education system, we began to iteratively adapt MHN into a framework for trainees in medicine; we presented the ideas at four different research-in-progress sessions where we collected input from individuals with expertise in medical education, adult learning theory, professional identity formation, conceptual models, and learner assessment [28]. We received input from educational administrators, educators, and trainees from a wide range of identities – with particular awareness to learn from those from historically marginalized groups. With their indispensable and extensive contributions, the authors ultimately came to consensus about how to adapt MHN and present the novel framework.

Many diverse elements, some intrinsic to the trainee and others inherent to the medical education system, influence the extent to which any individual will have their needs satisfied during their training [7,8]. Because we wanted to respect the breadth of individuality across medical trainees, and especially with our prioritization of those who come from historically marginalized groups, we considered the many inequities that may interfere with a trainee’s capacity to learn and succeed during their medical training [4,7,8].

## The Adapted Novel Framework that May Enhance the Support of Medical Trainees – Especially those from Historically Marginalized Groups Facing Structural Inequities

This revised framework for trainees in medicine retains all of the foundational needs from Maslow’s original theory (physiological, safety/security, belonging, and esteem). The literature, expert opinion, and our own discussions were harmonized and, there seemed to be face validity that all four needs are germane for medical trainees’ development [14,19,34,35,36,37]. Trainees in medicine who have these needs satisfied are more likely to realize socioemotional and cognitive growth in the learning environment [12].

**Figure 2:**
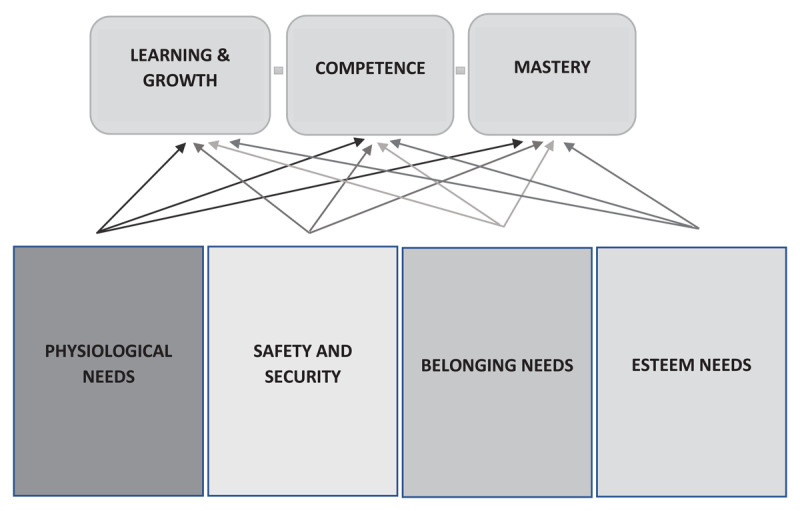
**Framework adapted from Maslow’s theory that illustrates how the needs of medical trainees relate to their success in realizing educational outcomes**. This conceptual framework was based on Maslow’s ‘Hierarchy of Needs’ and considers the needs of trainees within the medical education system. All of these needs are shown to be equally important. They can be concurrently met or unmet based on each trainee’s unique circumstances and experiences. The degree to which these needs are satisfied directly impacts the trainee’s capacity to ‘learn and grow’, attain ‘competence’, and progress toward ‘mastery’.

In adapting Maslow’s original theory for this medical education framework, the sequential order was intentionally removed; notably, the hierarchy has not been supported by previous research [38,39]. It was decided for the new framework that the four foundational needs were equally vital – in that each must be continuously satisfied for the success of medical trainees [12,27,28,29]. They have each been described to be necessary for meaningful learning and desirable academic outcomes among medical trainees [19,36,37]. Another change compared to the original MHN was the removal of ‘self-actualization’ at the pinnacle. This decision was based on the literature and with input as well as corroboration from our consultants; prior studies of Maslow’s theory have questioned self-actualization’s tangible existence and relevance [29,40]. In the novel framework for medical education, self-actualization is replaced by conclusive outcomes in medical education including ‘learning and growth’, attainment of ‘competence’, and continuous progress toward ‘mastery’ (Figure 2) [41,42,43,44]. Learning and growth is defined as the experience of acquiring knowledge that leads to changes in one’s understanding or behavior [35]. Competence is the building of knowledge, skills, and behaviors that allow a trainee to reach specific milestones in their professional development [42]. Mastery involves the integration of knowledge that allows adaptation to new clinical situations/contexts, consistently high performance, and the commitment to making steady progress [43].

In considering how best to schematically present the novel framework for medical education, we acknowledged that for some skills and behaviors in medicine, mastery is either impossible or unrealistic – and the goal should instead be continual learning and growth. Examples for this include areas such as professionalism and humanized caring for patients. As such, the framework is not represented as a pyramid that comes together at a singular target; instead, above the needs are the range of desired educational goals and outcomes.

## From Theory to Application: Practical Use of the Framework in UME and GME

Pragmatically, this framework for practical purposes highlights areas where trainees require support in order to achieve learning, growth, competence, and mastery across medical education systems. It also underscores the needs to be considered when a trainee is underperforming or struggling using an equity approach [41,42,43]. Trainees’ social identities can shape their experiences within the medical education system.

For illustrative purposes, below we demonstrate how the framework might be valuable for individual trainees and educational training programs to shift perception of underperformance from a deficit lens to one that considers equity [6,11,12].

### Practical application of the framework for individual trainees

Perhaps it is worth explicitly stating that if a trainee is truly flourishing and thriving within the medical education system, it may be safe to assume that their needs are largely being met [13]. By contrast, among those who are struggling, we are highlighting the notion that it is necessary to consider unmet needs as part of the root cause [14,34,35,36].

**Case example** – Baxter is a FGLI medical student confronting financial insecurity during Year 2 of medical school. In working with the framework independently and candidly, he will realize (and be able to explain to others) that his unmet physiological needs (hunger and fatigue) and safety needs (related to his living situation and commute) are impacting his journey in medical school. The framework explicitly emphasizes to Baxter that his unmet needs threaten his potential for learning, growth and attainment of competence (professional development). This reframing of Baxter’s hardship to their root causes might demonstrate that he is not likely to be able to remedy the situation entirely on his own. Given his determination and career goals, it is hoped that he would be moved to seek assistance. If this critical appraisal of needs were to happen more regularly, stigma might be lessened, and trainees like Baxter might feel more comfortable to coming forward for help [26].

We also hope that the framework may be used by clinician educators and course directors to reflect on their trainees’ needs and the structural barriers that undermine them.

**Case example** – Dr. Jones is a teaching-attending working with four 3^rd^ Year medical students on their internal medicine clerkship rotation. One of them, Harriet, is struggling substantively compared to her peers. She seems to be less knowledgeable, is not connecting as well with patients or the team, and she appears to be somewhat apathetic about learning. Dr. Jones might try harder to teach Harriet and make assumptions about her aptitudes. Alternatively, Dr. Jones might look to the framework and consider the extent to which Harriet’s needs are being met. During an interval feedback meeting to discuss performance, Dr. Jones can go over the framework with Harriet to explore the relationship between unmet needs and learning/growth. In this example, Harriet explained that she felt different from all other team members (students, residents, and attending) and that some comments on the early days of the rotation reinforced her ‘otherness’. Her unmet belonging needs were causing her to wilt and feel like an imposter. With this insight, Dr. Jones was able to support Harriet differently, tweak the learning environment with the whole team, and coach her with strategies for speaking up and managing such situations.

The framework can serve as a starting point for approaching trainees who are struggling – particularly if they are from a historically marginalized group. It can remind the educator to consider an equity lens; it may also facilitate a discussion of needs in a way that is both destigmatizing and conveys the consequences of unmet needs [1,37]. Without considering this framework, educators may fall back on a deficit-based appraisal when judging the trainee’s performance; this may lead to a deficit-based remediation strategies [25]. If brought into the discussion, the framework might encourage some trainees to share marginalized parts of their identity, such as first generation or low income status, and to ask for help [1,17,36,37]. We hope that if the framework is introduced into performance discussions between educators and trainees, the relationships may be strengthened, and these pairs could partner in supporting the trainee’s success [12,19,34,35,36]. Such consideration, including learner-centered remediation and of the social determinants of learning, will go a long way in making the learning environment more supportive and welcoming [45,46].

### Application of the framework for a training program

Currently, with a few exceptions, medical trainees go through the same training in the same amount of time, with the same programmatic supports; the medical education system treats them as a fairly homogenous cohort [1,2,3]. This is largely due to the fact that there are insufficient resources to allow for discerning the specific nuances of each trainee and elaborating an individualized educational approach for each of them. This new framework can assist educational programs and leaders in characterizing the extent to which their trainees are having their basic needs met [5,6,8,11,12,18,45]. In considering the framework (which we are in the process of converting into a validated tool where trainees assess both [i] whether their needs being met and [ii] the impact of these needs on educational outcomes), training programs can know the extent to which needs are met or unmet by all (or a subset of trainees from specific groups). These details could highlight blind spots within a program/institution. For instance, if the majority of students who self-identity as low income consistently report issues with physiological and/or safety/security needs, these details could be helpful for planning and reassessing the appropriateness of financial aid packages and the structural resources made available for trainees [47].

## Limitations

Several limitations of the proposed framework should be considered. First, while it covers the breadth of needs that are highly relevant for most trainees, there may be some needs that are not acknowledged or accounted for. Given the uniqueness of each trainee and particularly the myriad inequities faced by trainees from historically marginalized groups, it would not be prudent to suggest that any framework would comprehensively cover the needs of every trainee. Second, complex factors such as time and financial resources are not explicitly depicted in the framework; instead, these were considered to be intricately embedded within other needs. Finally, the authors have considered how this framework could be applied to multiple trainee identities and scenarios (especially trainees from historically marginalized groups), but it is unclear if it will be suitable across all combinations of identities and experiences. The framework’s generalizability will be systematically assessed in our ongoing studies as we develop a tool based on the framework and as we establish additional validity evidence.

## Conclusions

The training involved to become a physician is onerous for all. To succeed and flourish in the medical education system, trainees must have their needs met so they can learn and grow. For trainees who struggle, deficit-based discourse and related interventions may do them a disservice because they do not fully explore or address the root causes – including the structural barriers and inequities that exist within the medical education systems.

Many trainees from historically marginalized groups disproportionally have unmet needs compared to their non-marginalized peers; this is in large part due to experienced inequities and the additional emotional, physical, mental, and cognitive burdens they sometimes carry. The novel framework described in this paper, adapted from Maslow, counters the deficit model. It shows how medical trainees’ unmet needs represent structural inequities that can contribute to struggling and failure within the medical education system. This framework will hopefully prompt trainees to regularly consider their needs throughout medical training; it should be empowering for them to corroborate what they are experiencing and give them shared terminology to describe needs that are not being met. Trainees from historically marginalized groups who are underperforming or struggling within the medical education system may benefit most from the framework – particularly if they seek out and find support to better fulfill their needs. The framework might compel training programs and institutions to consider the attention they devote to their trainees’ needs and their allocation of supportive resources. Ideally, medical education systems will become a more equitable environment where all trainees can thrive.
